# The Role of microRNA in Overactive Bladder: Relationship and Clinical Correlation †

**DOI:** 10.3390/medicina61030475

**Published:** 2025-03-08

**Authors:** Kürşat Küçüker, Hülya Aybek, Hakan Akça, Ege Rıza Karagür, Elif Fırat, Yusuf Özlülerden, Sinan Çelen, Zafer Aybek

**Affiliations:** 1 Department of Urology, Pamukkale University School of Medicine, Denizli 20070, Turkey; 2 Department of Medical Biochemistry, Pamukkale University School of Medicine, Denizli 20070, Turkey; 3 Department of Medical Genetics, Pamukkale University School of Medicine, Denizli 20070, Turkey; 4 Department of Medical Biochemistry, Manisa Celal Bayar University School of Medicine, Manisa 45140, Turkey

**Keywords:** overactive bladder, microRNA, biomarker, cholinergic

## Abstract

*Background and Objectives*: This study aimed to determine the relationship between miRNAs and overactive bladder (OAB). We also aimed to reveal the diagnostic properties of miRNAs and their potential to predict responses to therapy. *Materials and Methods:* The study included 60 patients with OAB as the treatment group and 60 healthy individuals as the control group. The blood levels of 15 miRNAs in both groups were determined using PCR. Also, miRNAs with high diagnostic values were identified with receiver operating characteristic (ROC) curves. Finally, the Turkish-validated OAB questionnaire form was filled out before and after the treatment by the participants in the treatment group. In this way, the relationship between OAB score changes and miRNA levels was examined. *Results:* The let-7a, let-7c, let-7e, let-7f, and let-7g miRNA molecules in the treatment group were higher, with a high level of significance (*p* = 0.0001). Additionally, the miR-135b, miR-300, miR-372, miR-373, miR-381, miR-520a, miR-520d, and miR-520e miRNA molecules were found to be statistically higher in the control group (*p* = 0.0001). In addition, let-7c (area under curve [AUC] = 0.985) and the let-7c + miR-381 combination (area under curve [AUC] = 1) were the highest values in the ROC analysis. Finally, after treatment in the patient group, a significant difference was detected in both miRNAs (let-7f and miR-135b) in patients with clinical improvements of 50% and above in the OAB score. *Conclusions:* miRNAs may help elucidate the pathophysiology of OAB. They may shed light on diagnosis and evaluation of treatment effectiveness.

## 1. Introduction

Overactive bladder (OAB) is a common condition among older men and women and can significantly reduce quality of life. OAB is defined as a syndrome by the International Continence Society (ICS) and is characterized by urine urgency, with or without urgent urinary incontinence, typically with increased daytime frequency and nocturia, provided that there is no established infection or other evident disease. Globally, the prevalence of OAB is currently 11.9% in women and 10% in men, with an average frequency of 10.9% [1]. In a study conducted in Turkey, OAB was reported as 20% in men and 35.7% in women [2].

The pathophysiology of OAB is not fully understood and is thought to have a multifactorial etiology [3]. Muscarinic cholinergic receptors primarily mediate bladder contraction, while adrenergic receptors facilitate relaxation, ensuring proper coordination of bladder function [4]. Therefore, clinicians prefer mostly muscarinic receptor antagonists for the medical treatment of OAB; however, medical treatment of OAB often does not provide sufficient benefit and muscarinic receptor antagonists even cause significant side effects in some patients, leading to treatment discontinuation. Although many biomarkers have been evaluated to predict treatment success, they have not been applied for routine clinical use [5,6].

microRNAs (miRNAs), short RNA molecules of 20–22 nucleotides, regulate genes post-transcriptionally. miRNAs are transferred to the cytoplasm after being synthesized in the nucleus and bind to mRNA, regulating protein synthesis and gene expression through the suppression of translation or cleavage of mRNA according to the level of coupling. Lawrie et al. suggested using microRNAs (miRNAs) as non-invasive biomarkers to detect, treat, and predict bladder disorders, prostate disease, and cancer [6,7].

Rho-related kinase (ROK/ROCK) and guanine nucleotide exchange factors (GEFs) are prominent proteins within the cholinergic pathway, which plays a significant role in the regulation of voiding physiology. Dysregulation of these proteins causes an excessive contractile response of bladder smooth muscle [4]. *ADRB3* which encodes the beta 3-adrenergic receptor, also plays a role in bladder activity as hypo-functioning of these receptors causes detrusor muscle dysfunction and impaired urinary function. Also, Ferreira et al. demonstrated that urinary dysfunction in patients with OAB was associated with *ADRB3* polymorphisms [8]. Atayi et al. identified miR-92a-3p, miR-146a-5p, and miR-491-5p as potential biomarkers for benign urological conditions such as OABs and BOO (bladder outlet obstruction). Their findings highlight the role of miRNAs in early diagnosis and disease monitoring, reinforcing their significance in bladder dysfunction research [9].

miRNAs modulate bladder function by targeting key genes involved in adrenergic and cholinergic signaling pathways, altering mRNA stability and protein translation. In the adrenergic pathway, miRNAs regulate *ADRB3* expression, which encodes the β3-adrenergic receptor responsible for detrusor relaxation via cyclic AMP (cAMP) activation of protein kinase A (PKA). By influencing *ADRB3* levels, miRNAs may alter cAMP-mediated smooth muscle relaxation, affecting bladder storage function [9]. In the cholinergic pathway, miRNAs, often referred to as “cholinomiRs”, regulate genes encoding muscarinic receptors (M2 and M3), which control detrusor contractions through the G-protein-coupled activation of phospholipase C (PLC) and intracellular calcium release. Additionally, miRNAs targeting *ROCK2* and *ARHGEF10*, critical components of the *Rho/ROCK* pathway, may modulate calcium sensitivity and smooth muscle contractility, further contributing to the pathophysiology of OAB [10].

We investigated miRNAs’ relationships to ROCK2, ARHGEF10, and ADRB3; the first two encode the cholinergic pathway proteins ROCK2 and RhoGEF, respectively. Our aim was to try to understand the pathogenesis of OAB by examining the relationship between miRNAs and their target genes. Although it is difficult to explain the pathogenesis with miRNAs alone, since there are multiple factors in the formation of OAB, miRNAs may be useful in explaining the pathogenesis at the molecular level. We aimed to see whether the miRNA values were different in the patient and control groups. We also aimed to see whether there is a relationship between miRNA values of patients whose OAB symptom scores improved and did not improve with treatment. In this way, we aimed to predict the outcome of medical treatment among those who suffer from OAB.

## 2. Materials and Methods

### 2.1. Patient and Healthy Group Selection

After approval from the Pamukkale University Faculty Medicine Ethics Committee, dated 26 June 2018 and numbered 13, we included 120 women over the age of 18 in our study and divided them into two groups: 60 were the patients diagnosed with OAB for the first time and 60 were healthy individuals. We examined the patients for differential diagnosis. We performed urinalysis, urine culture, serum creatinine, serum CRP levels, and urinary ultrasonography. Each participant in the study successfully completed a three-day voiding diary. The OAB-V8 questionnaire (validated Turkish version) was completed by all the individuals [11]. OAB-V8 is preferred due to its ease of use in clinical practice. In the patient group, we showed that there was no bladder outlet obstruction by performing a pressure-flow urodynamic study in patients with a peak flow rate of less than 10 mL/s in uroflowmetry values. Idiopathic overactive bladder was diagnosed if patients reported symptoms of problematic urgency or a compelling desire to void, scored at least 8 points on the OAB-V8 questionnaire, and did not fulfill any exclusion criteria. The study involved excluding patients who came with neurogenic bladder, bladder outlet obstruction, and urinary tract disorders such as stones, tumors, and infections. We selected patients without urinary stress incontinence. A control group consisting of hospital staff and their family who were matched in terms of age and sex was selected. These individuals did not exhibit any urinary system issues. Prior to the study, none of the participants had received a diagnosis or undergone treatment for an overactive bladder. No anticholinergic drug is clearly superior to another for cure or improvement of OAB, so we selected the standard 5 mg dose of solifenacin for treating OAB. After one month of treatment, the patients were reassessed. A 50% or greater reduction in the OAB symptom score was considered as a treatment success.

### 2.2. RNA Extraction

Venous blood was collected from all participants in 2 tubes with 2 mL EDTA, one for DNA isolation and the other to separate RNA. To minimize the impact of diurnal variations on miRNA expression, all blood samples were collected between 10:00 AM and 12:00 PM, ensuring consistency across all participants. Additionally, samples were obtained after an overnight fast, reducing potential variability due to dietary influences. After these samples were centrifuged to obtain plasma, The miRNAs were isolated utilizing the miRNeasy Serum/Plasma Advanced Kit according to the manufacturer’s instructions (QIAGEN, Hilden, Germany). Total RNA concentration and purity were determined by measuring A260/280 and A260/230 ratios with the Nanodrop device (Nano, Maestrogen, Hsinchu, Taiwan). Samples with a 260/280 ratio of 2.0 and close to 2.0 were included in the study. RNA samples were stored at −80 °C to maintain stability until the study was performed.

### 2.3. Selection of miRNAs

**Figure 1:**
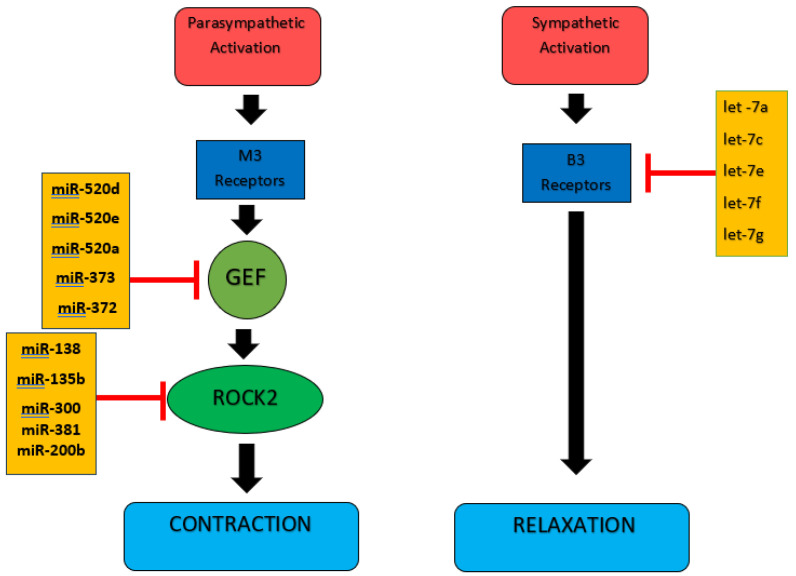
The selected miRNAs, the genes they affect, and the hypothesis.

We selected miRNAs that target ADRB3, ARHGEF10, and ROCK2 using TargetScan, miRTarBase, miRDB, and microRNA.org databases. Instead of focusing on previously studied miRNAs, we aimed to explore less-investigated miRNA candidates that may also regulate these genes, acknowledging that a single gene can be influenced by multiple miRNAs. By selecting miRNAs consistently predicted in all four databases, we increased the specificity of our analysis while broadening the scope of research on miRNA-mediated regulation in OAB. According to the data, let-7a, let-7c, let-7e, let-7f, and let-7g for *ADRB3*; miR-138, miR-135b, miR-300, miR-381, and miR-200b for *ROCK2*; and miR-520d, miR-520e, miR-520a, miR-373, and miR-372 for *ARHGEF10* were selected. The selected miRNAs, the genes they affect, and the hypothesis are shown in Figure 1.

### 2.4. Measurements of miRNA Expression Levels

During the validation stage, a quantitative polymerase chain reaction (qPCR) analysis was conducted using the miRCURY LNA SYBR Green PCR Kit (QIAGEN, Hilden, Germany) on the StepOnePlus Real-Time PCR (Applied Biosystems, Thermo Fisher Scientific, Waltham, MA, USA), following the procedures provided by the manufacturers. miRCURY LNATM miRNA PCR Assays (QIAGEN, Hilden, Germany) were used as RNA primers. The data were processed using the StepOnePlus Real-Time PCR software v2.3 (Applied Biosystems, Thermo Fisher Scientific, Waltham, MA, USA). miRNA expression levels were normalized to that of miR-30e-5p. At the end of the analysis, for each miRNA whose expression could be detected, the cycle threshold value (the number of cycles in which the miRNA expression amount exceeded the threshold value) was obtained. In all samples, the ∆ct (∆ct = Ct value of related gene—Ct value of normalizer gene) value was calculated for each gene separately. miRNA expression values were obtained by performing the 2^Δ*C*tOAB−Δ*C*t Normal^ formula. Preprocessing data by averaging the median normalization and logarithmic transformation is the most used miRNA normalization approach. The results of the two groups are given as fold changes. 

### 2.5. Statistical Analyses

The statistical data analysis was performed using SPSS v24.0 (IBM, Chicago, IL, USA). In this study, numerical data such as continuous variables, means, standard deviations, maxima, minima, medians, and categorical variables are represented using numerical values and percentages. The Mann–Whitney U test was utilized to assess variations among the independent groups, given that the parametric test assumptions were satisfied. Pearson correlation analysis and chi-square analysis were used to compare continuous and categorical variables. Also, receiver operator characteristic (ROC) curves were used for diagnostic performance analyses of variables. Statistical significance was defined as *p* < 0.05 in all analyses.

## 3. Results

The treatment and control groups averaged 56.5 and 55.8 years old. Height averaged 156.7 cm in the treatment group and 158.8 cm in the control group. The treatment group’s weight averaged 74.0 kg, the whereas control group’s weight averaged 72.0 kg. The treatment group had an average BMI of 30.5 kg/m^2^ and the control group had 28.6 kg/m^2^. No significant difference was identified in age, height, weight, or BMI across groups. The treatment and control groups’ demographics are shown in Table 1.

Urinary incontinence was observed in 54 of the total 60 patients within the treatment group. It was observed that urinary incontinence in 18 patients improved after 1 month of treatment with solifenacin. The urinary incontinence status of the patient group before and after treatment is demonstrated in Table 2.

In the treatment group, 12 out of 60 patients had an improvement of at least 50% or more in their OAB symptom scores. One out of twelve patients did not report any incontinence before or after treatment. The remaining 11 patients described incontinence before and after treatment.

The treatment group exhibited significantly greater expression levels (median [range]) of let-7a (6.8 [0.02–97.7]), let-7c (23.1 [0.31–260]), let-7e (7.42 [0.34–44.01]), let-7f (40.9 [0.03–485]), and let-7g (17.75 [0.44–855]) compared to the control group. The treatment group had significantly lower levels of miR-300 (2.23 [0.14–11.6]), miR-381 (33.18 [5.16–446]), miR-373 (4.54 [0.02–61.2]), miR-372 (5.06 [0.1–49.78]), miR-520a (0.7 [0.01–9.13]), miR-520d (2.59 [0.03–72.9]), miR-135b (0.36 [0.04–79.6]), and miR-520e (3.27 [0.14–19.5]) compared to the treatment group. Nevertheless, no statistically significant distinction was observed in the expression levels of miR-138 and miR-200b between the two groups. The miRNA expression levels are offered in Table 3.

We looked at the ROC curves of all the miRNAs to see how useful they were for diagnosis. let-7c, let-7e, miR-300, and miR-381 had the highest AUC values, which were 0.985, 0.939, 0.912, and 0.954, in that order. Specificity and sensitivity were 98.3% and 94.6% for let-7c. In the case of let-7e, sensitivity was 98.2% and specificity was 77.6%. For miR-300, sensitivity was 77.3% and specificity was 95.6%. In the case of miR-381, sensitivity was 87.0% and specificity was 89.7%.

let-7c regulates *ADRB3*, a key component in β3-adrenergic signaling involved in detrusor muscle relaxation. miR-381 targets *ROCK2*, a critical regulator of the *Rho/ROCK* pathway, which controls smooth muscle contractility. Their strong diagnostic performance suggests they may play a role in OAB pathophysiology by modulating adrenergic and contractile signaling pathways.

The sensitivity and specificity of miRNA combinations were also examined. The highest AUC miRNAs were merged, and their AUC values were calculated. The let-7c + miR-381 combination had 100% specificity and sensitivity with an AUC of 1. The let-7f + let-7c combination had a 0.991 AUC, 96.2% sensitivity, and 98.2% specificity. The let-7f + let-7e combination had a 0.944 AUC, 80.0% sensitivity, and 96.5% specificity. The let-7f + miR-135b combination had a 0.886 AUC, 78.1% sensitivity, and 90.6% specificity. The miR-373 + let-7c combination had a 0.995 AUC, 98.2% sensitivity, and 96.4% specificity. Table 4 shows ROC curve, AUC, and *p* values for different miRNAs and combinations.

The OAB-V8 questionnaire score was 29.2 in the treatment group before treatment with solifenacin, and it decreased to 21.3 after 1 month of treatment. OAB-V8 scores before and after treatment in the treatment group are offered in Table 2.

Patients in the treatment success and failure groups were compared for miRNA expression. The results of two miRNAs were statistically significant. let-7f was 147.86 (0.06–484.38) in the treatment success group, while it was 32 (0.03–426.91) in the treatment failure group (*p* = 0.045). miR-135b was 0.06 (0.03–0.21) in the treatment success group, while it was 0.3 (0.01–14.32) in the treatment failure group (*p* = 0.036). No substantial difference was seen in other miRNAs’ expression. The changes in miRNA expression levels are presented in Table 5.

**Figure 2:**
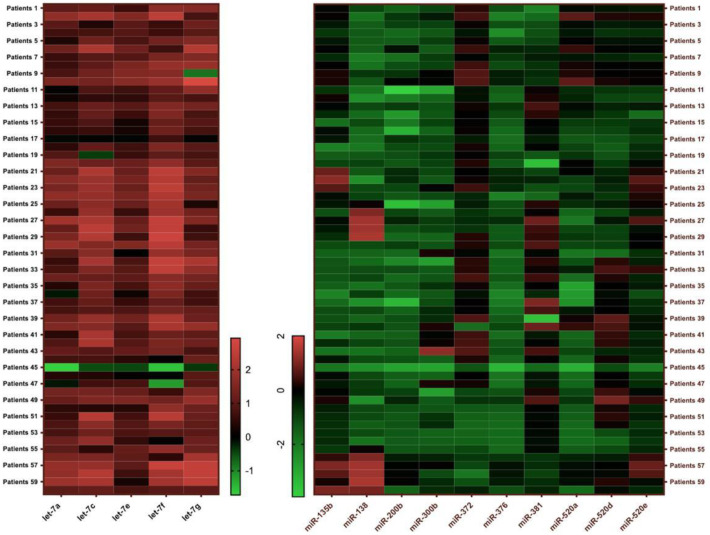
The expression patterns of differentially expressed miRNAs between the OAB and control groups.

A heatmap was generated to visualize the expression patterns of differentially expressed miRNAs between the OAB and control groups (Figure 2). The heatmap illustrates distinct clustering patterns, with several miRNAs exhibiting higher or lower expression levels across groups, further supporting their potential role as biomarkers for OAB.

## 4. Discussion

Our study found that the expression levels of let-7a, let-7c, let-7e, let-7f, and let-7g, which target *ADRB3*, were significantly higher in patients with OAB compared to the control group. This suggests these miRNAs may contribute to OAB symptoms by inhibiting *ADRB3*, reducing β3-adrenergic receptor activation, and impairing detrusor relaxation. Similarly, the targeting activity of miR-135b, miR-300, miR-381, miR-376, miR-372, miR-520a, miR-520d, and miR-520e against *ROCK2* and *ARGHEF10* was significantly higher in the control group than in the treatment group. Given that *ROCK2* and *ARGHEF10* are involved in *Rho/ROCK* pathway activation, which increases smooth muscle contractility, their dysregulation may contribute to detrusor overactivity and OAB pathophysiology. The simultaneous suppression of *ADRB3*-driven relaxation and the upregulation of *ROCK2-/ARGHEF10*-driven contraction may collectively drive OAB symptoms by disrupting the balance between detrusor muscle contraction and relaxation. These findings emphasize the importance of miRNA-mediated regulatory mechanisms in OAB and their potential as diagnostic markers and therapeutic targets (Figure 1).

The ROC curve, also known as the susceptibility curve, is generally considered in the evaluation of diagnostic tests. It is also an indicator which reflects the sensitivity and specificity of variables. The AUC value is proportional to the diagnostic potential of a test [12]. The AUC values obtained in two previous studies on the OAB symptom score were 0.84 and 0.78, respectively [13,14]. AUC values were evaluated to identify disease biomarkers. In previous studies, AUC values for ATP, nerve growth factor, and brain-derived neurotrophic factor in OAB patients were 0.741, 0.741, and 0.780, respectively [15,16]. In our study, the AUC values for let-7c, let-7e, miR-300, and miR-381 were the highest and were 0.985, 0.939, 0.912, and 0.954, respectively. AUC values for the miRNA combinations of let-7c + miR-381, let-7f + let-7c, let-7f + let-7e, let-7f + miR-135b, and miR-373 + let-7c were 1, 0.991, 0.944, 0.886, and 0.995, respectively. According to these results, these miRNAs and miRNA combinations have high diagnostic adequacy for OAB which must be further evaluated. Although ROC analysis provides valuable insights into the diagnostic performance of miRNAs, it alone is insufficient for biomarker validation. Validating independent cohorts is essential to confirm reproducibility and clinical applicability. Future studies should focus on validating these miRNAs in external patient cohorts to enhance their potential as diagnostic biomarkers for OAB.

miRNAs, which were initially investigated in various cancers, can be used to elucidate the pathophysiology of many different diseases. miRNAs are active in many biological processes including the immune response and cell death, development, proliferation, differentiation, and metabolism. miRNAs, by binding to mRNAs in the posttranscriptional stage, either inhibit or degrade these mRNAs and prevent their translation into proteins [17]. Zhang et al. reported increased miRNA levels in bladder samples of OAB patients [18]. They specifically found that miR-34a levels increased in the bladder samples and concluded that this miRNA increased the predisposition to OAB by acting in the cholinergic signaling pathway. In a similar vein, Kashyap et al. found that miR-132 and miR-221 expression increased in rat OAB models and concluded that these miRNAs induced bladder smooth muscle hypertrophy and detrusor dysfunction [19]. Also, Chermansky et al. found that miR-221 and miR-125b expression levels were upregulated in patients exhibiting OAB symptoms [20]. In addition, Cordes et al. reported that miR-143 and miR-145 affected smooth muscle tonus, while Imamura et al. reported that miR-1, which inhibits bladder smooth muscle contractility, may be a new treatment target in OAB patients [21,22]. Firat et al. investigated the relationship between various miRNAs and OAB. The authors found that the expression levels of let-7b, miR-92a, miR-98, miR-142, and miR-200c were significantly upregulated, whereas that of miR-139 was significantly downregulated, in blood samples from OAB patients [23]. Finally, Atayi et al. identified miR-92a-3p, miR-146a-5p, and miR-491-5p as potential biomarkers for benign urological conditions such as an OAB and BOO. Their findings highlight the role of miRNAs in early diagnosis and disease monitoring, reinforcing their significance in bladder dysfunction research [9]. These findings support the claim that increased miRNA expression levels affect the regulation of bladder smooth muscle contraction. However, unlike our study, there was no significant relationship between OAB symptom scores and miRNA expressions. In our study, we focused on the relationship between the change in OAB symptom scores and miRNA levels. In addition, the miRNAs we chose were not included in their research. Since there is no clear relationship between target genes and miRNAs affecting these genes, we planned to work with different miRNAs.

The OAB-V8 questionnaire was also employed to evaluate the severity of OAB and the treatment response in terms of symptom scores. This questionnaire allows a simple and reliable assessment of symptoms and treatment efficacy in daily clinical practice, unlike other tests which are complex and difficult [11,12,13,14,15,16,24]. Before and after 1 month of solifenacin treatment, we evaluated the symptom scores and investigated the relationship between changes in these scores and miRNA expression levels. In terms of let-7f and miR-135b, the change in the levels of these miRNAs in the patients with a 50% or greater reduction in the OAB V-8 score after treatment was significantly different from the change in the patients who did not respond to the treatment or had a lower rate of change. Thus, it may be possible to make predictions about treatment effectiveness by measuring these two miRNAs, let-7f and miR-135b, before medical treatment in OAB patients.

In our study, the functional relevance of miRNA alterations was explored by comparing OAB-V8 scores before and after treatment, assessing whether changes in miRNA expression correlated with symptom improvement. This clinical approach provides valuable insights into their potential role in OAB pathophysiology. However, future studies should incorporate functional experiments in the bladder’s smooth muscle cells, such as knockdown, overexpression, and luciferase reporter assays, to further validate their mechanistic effects.

To date, there are still uncertainties regarding the pathophysiology of OAB. Currently, questionnaires are the most frequently employed tools for diagnosing and determining patients’ responses to the treatment of OAB. Thus, the search for different molecular markers for OAB continues. In this study, we aimed to determine whether miRNAs can serve as biomarkers in the diagnosis of OAB and whether they can evaluate the effectiveness of treatment before treatment in patients with OAB.

This study has limitations. We started with female patients. Future investigations with male patients are encouraged to confirm and expand our findings. We think this study helps improve OAB treatment techniques and biomarkers. However, miRNA screening plus pre-treatment blood analysis can predict therapy response and minimize unnecessary usage of muscarinic receptor antagonists. Future research should include additional patients, measure and compare miRNA levels before and after treatment, and include other polymorphisms to corroborate these findings.

Although OAB diagnosis primarily relies on symptom-based methods and urodynamic studies, miRNA-based diagnostics may provide a minimally invasive and rapid alternative. While qPCR-based miRNA tests are becoming more cost-effective, clinical implementation requires standardization, large-scale validation, and rapid testing technologies. Future studies should focus on optimizing these aspects to enable the clinical use of miRNA diagnostics for OAB.

This study has several limitations. First, the study cohort consisted exclusively of female patients. Future research should include male patients to confirm and expand the generalizability of our findings. Despite the potential of miRNA screening combined with pre-treatment blood analysis in predicting therapy response and minimizing the unnecessary use of muscarinic receptor antagonists, additional validation is required. Although we believe that a one-month follow-up is adequate given the rapid onset of anticholinergic effects, long-term follow-up data are essential to assess the stability of miRNA-based biomarkers and their sustained predictive value in OAB treatment. Future studies should incorporate larger patient cohorts, evaluate miRNA levels before and after treatment, and consider additional genetic polymorphisms to strengthen these findings. Furthermore, functional validation studies, including in vitro investigations of miRNA–mRNA interactions, are necessary to elucidate the mechanistic role of these miRNAs in OAB pathophysiology. The potential influence of genetic polymorphisms and comorbidities on miRNA expression was not specifically addressed in our study and should be considered in future research. Additionally, while our study identified miRNA alterations associated with OAB, it did not determine whether these changes are specific to OAB or shared with other lower urinary tract disorders. Comparative analyses across OAB, bladder outlet obstruction (BOO), neurogenic bladder dysfunction, and interstitial cystitis are necessary to establish whether these miRNAs serve as specific diagnostic biomarkers for OAB or reflect broader bladder dysfunction mechanisms. Another limitation is the exclusive use of blood-based miRNA analysis, which may impact bladder specificity. The incorporation of urinary and bladder tissue samples in future studies will be critical for confirming their diagnostic potential and improving the reliability of miRNA-based biomarkers for OAB.

## 5. Conclusions

OAB is a symptom-based syndrome with undetermined etiopathogenesis. Currently, treatment can only reduce symptoms instead of curing the disease. These findings may help us better understand etiopathogenesis. In this study, we discovered a notable increase in the expression levels of miRNAs that affect *ADRB3* in the patient group compared to the healthy group, suggesting that the increased expression of these miRNAs can cause the onset of OAB symptoms by inhibiting *ADRB3*. The expression levels of miRNAs involved in the cholinergic pathway were lower in the patient group than in the healthy group, and a decrease in the expression levels of these miRNAs led to a decrease in the inhibitory effect on the cholinergic pathway and related genes, suggesting that this leads to OAB symptoms. Through extensive studies, researchers can better understand the interaction between miRNAs and the cholinergic and adrenergic pathways to develop specific treatment strategies. In patients who experienced a 50% or greater decrease in the OAB score, the expression level of let-7f, which targets ADRB3, was higher than that of the group with no change or a lower rate of OAB score reduction. In addition, miR-135b, which targets *ROCK2*, was higher in the group with no or a lower rate of OAB score reduction. These findings can be supported by additional studies to predict treatment success before treatment.

## Figures and Tables

**Table 1:** Demographic data of patient and control groups.

	Patient Group (*n* = 60)		Control Group (*n* = 60)		
	Mean ± SD	Median	Mean ± SD	Median	*p*
Height (cm)	156.67 ± 6.26	155.5 (139–170)	158.78 ± 6.74	160 (140–172)	0.079
Weight (kg)	73.97 ± 16.4	73.5 (40–125)	72.02 ± 13.01	70 (50–112)	0.472
BMI (kg/m^2^)	30.04 ± 6.17	30.28 (17.78–45.91)	28.58 ± 5.05	28.11 (19.2–43.75)	0.101
Age (years)	56.48 ± 15.08	60 (23–83)	55.82 ± 11.83	58.5 (27–76)	0.567

**Table 2:** Urinary incontinence status of the patient group before and after treatment.

	Urinary İncontinence	No Urinary İncontinence	*p*
Before treatment	54	6	0.0001 *
After treatment	36	24	

*: statictically significant.

**Table 3:** Expression levels of miRNAs in patient and control groups (2^−∆Ct^).

	Patient Group (*n* = 60)	Control Group (*n* = 60)	*p*
let-7a	6.8 (0.02–97.68) (*n* = 58)	1.96 (0.11–78.25) (*n* = 60)	0.0001 *
let-7c	23.1 (0.31–259.57) (*n* = 55)	0.19 (0–2.3) (*n* = 57)	0.0001 *
let-7e	7.42 (0.34–44.08) (*n* = 54)	0.14 (0–9.61) (*n* = 58)	0.0001 *
let-7f	40.93 (0.03–484.38) (*n* = 54)	1.5 (0.13–35.75) (*n* = 58)	0.0001 *
let-7g	17.75 (0.44–855.13) (*n* = 55)	5.37 (0.12–92.41) (*n* = 56)	0.0001 *
miR-135b	0.24 (0.01–14.32) (*n* = 44)	0.36 (0.04–79.58) (*n* = 55)	0.049 *
miR-138	0.04 (0–29.56) (*n* = 17)	0.05 (0–1.8) (*n* = 11)	0.557
miR-200b	0.04 (0–0.97) (*n* = 44)	0.07 (0–1.24) (*n* = 36)	0.157
miR-300	0.06 (0–15.06) (*n* = 22)	2.23 (0.14–11.63) (*n* = 45)	0.0001 *
miR-381	2.08 (0–29.74) (*n* = 54)	33.18 (5.16–446.39) (*n* = 58)	0.0001 *
miR-373	0.04 (0–0.5) (*n* = 51)	4.54 (0.02–61.18) (*n* = 58)	0.0001 *
miR-372	1.89 (0.02–69.36) (*n* = 57)	5.06 (0.1–49.77) (*n* = 59)	0.0001 *
miR-520a	0.13 (0–5.42) (*n* = 58)	0.7 (0.01–9.13) (*n* = 55)	0.0001 *
miR-520d	0.45 (0.02–9.21) (*n* = 58)	2.59 (0.03–72.86) (*n* = 58)	0.0001 *
miR-520e	0.38 (0.01–6.74) (*n* = 58)	3.27 (0.14–19.53) (*n* = 57)	0.0001 *

*: statictically significant.

**Table 4:** AUC (area under curve) and *p* values of miRNAs and miRNA combinations according to ROC curves.

	AUC	*p*
let-7a	0.767	0.0001 *
let-7c	0.985	0.0001 *
let-7e	0.939	0.0001 *
let-7f	0.876	0.0001 *
let-7g	0.714	0.0001 *
miR-135b	0.615	0.049 *
miR-138	0.433	0.557
miR-200b	0.592	0.157
miR-300	0.912	0.0001 *
miR-372	0.709	0.0001 *
miR-373	0.880	0.0001 *
miR-381	0.954	0.0001 *
miR-520a	0.727	0.0001 *
miR-520d	0.789	0.0001 *
miR-520e	0.861	0.0001 *
let-7c + miR-381	1	0.0001 *
let-7f + let-7c	0.991	0.0001 *
let-7f + let-7e	0.944	0.0001 *
let-7f + miR-135b	0.886	0.0001 *
miR-373 + let-7c	0.995	0.0001 *

*: statictically significant.

**Table 5:** miRNA (let-7f, mir-135b) expression levels in treatment success and treatment failure groups.

	Treatment Failure (Did Not Respond Clinically to Treatment or Less Than 50% Reduction in OAB V-8 Score After Treatment)	Treatment Success (50% or Greater Reduction in the OAB V-8 Score After Treatment)	*p*
*n*: 60	48	12	
let-7f expression levels	32 (0.03–426.91)	147.86 (0.06–484.38)	0.045 *
miR-135b expression levels	0.3 (0.01–14.32)	0.06 (0.03–0.21)	0.036 *

*: statictically significant.

## Data Availability

The data that support the findings of this study are available on request from the corresponding author. The data are not publicly available due to privacy or ethical restrictions.

## Abbreviations

OAB	Overactive bladder
ICS	International Continence Society
ROK/ROCK	Rho-related kinase
GEFs	Guanine nucleotide exchange factors
qPCR	Quantitative polymerase chain reaction
ROC	Receiver operator characteristic
AUC	Area under curve
